# The Predictive Power of Spatial Relational Reasoning Models: A New Evaluation Approach

**DOI:** 10.3389/fpsyg.2021.626292

**Published:** 2021-10-13

**Authors:** Marco Ragni, Daniel Brand, Nicolas Riesterer

**Affiliations:** ^1^Department of Computer Science, University of Freiburg, Freiburg, Germany; ^2^Predictive Analytics, Technical University Chemnitz, Chemnitz, Germany

**Keywords:** spatial cognition, cognitive models, individual human reasoner, mental model, predictive task

## Abstract

In the last few decades, cognitive theories for explaining human spatial relational reasoning have increased. Few of these theories have been implemented as computational models, however, even fewer have been compared computationally to each other. A computational model comparison requires, among other things, a still missing quantitative benchmark of core spatial relational reasoning problems. By presenting a new evaluation approach, this paper addresses: (1) developing a benchmark including raw data of participants, (2) reimplementation, adaptation, and extension of existing cognitive models to predict individual responses, and (3) a thorough evaluation of the cognitive models on the benchmark data. The paper shifts the research focus of cognitive modeling from reproducing aggregated response patterns toward assessing the predictive power of models for the individual reasoner. It demonstrate that not all psychological effects can discern theories. We discuss implications for modeling spatial relational reasoning.

## 1. Introduction

Please read the following two assertions and draw an inference (preferably without using any map):

**Table:** 

(1) Frankfurt is south-west of Amsterdam.
Amsterdam is north-east of Paris.
What is the spatial relation between Frankfurt and Paris?

*You can't say anything about the relation*, given only the above information. In the process of finding an answer to this question you may have formed a mental model of the cities according to the relations in your mind. In a second step, you may have inspected your mental model and have possibly searched for alternative models.

Cognitive psychology provides theories specifying such mental processes. Core predictions of these cognitive theories are tested experimentally, i.e., if participants in psychological experiments demonstrate the predicted behavior by the theories. Example 1 above is a so-called *indeterminate problem*, as no conclusion with a single cardinal relation between the cities Frankfurt and Paris can be determined by reasoning about the premises only. A *determinate problem* in contrast is for instance:

**Table:** 

(2) Frankfurt is south-east of Berlin.
Berlin is south-east of Stockholm.
What is the spatial relation between Frankfurt and Stockholm?

Because you can derive from the premises the conclusion “Frankfurt is south-east of Stockholm.”

Theories such as the theory of mental models (e.g., Johnson-Laird and Byrne, 1991) or the preferred mental model theory (Ragni and Knauff, 2013) predict that indeterminate problems (1) are more difficult than determinate problems (2). This depends on the underlying cognitive processes in both theories: the theory of mental models explains this effect by the need to construct more models in the indeterminate case and the theory of preferred models by the need to construct and vary a preferred model and its associated costs in the computational model PRISM. Such theoretically derived predictions are evaluated experimentally, i.e., the theoretical prediction holds, if there is a statistically significant difference in error rates between indeterminate and determinate problems. The statistical analysis often aggregates across both, problems and participants. Psychological experiments have been, conducted to test predicted effects such as the figural effect, the continuity effect, and the preference effect (Knauff et al., 1998; Ragni and Knauff, 2013).

Can we, however, assume that these effects and cognitive processes—that are supported by group-level data aggregation—do generalize to the level of individuals or is it possible that they cannot be observed in individuals (cf., Kievit et al., 2016; Fisher et al., 2018)? There is a potential danger, unless shown otherwise, that computational models do model well an effect supported by aggregating responses, which is not observable in any individual's reasoning process. At the same time an effect that can be identified in some individuals might not be visible in the aggregation.

Applied to the spatial problem above, general cognitive processes such as forming, inspecting, and searching for alternative models might be employed by most reasoners. However, there might be substantial differences between participant's in the way *how* they form their model and *how* they search for alternative models. We argue that cognitive theories need to focus on predicting an individual's response, as this can avoid many of the group-to-individual problems we have described above. But, to assess theories, data sets can be differ. So a second question is, are some data sets more suitable than others to test the predictive power of computational models? Hence, the goal of this paper is to comprehensively assess the predictive power of spatial relational reasoning models for the individual reasoner and to provide an assessment of the usability of existing data.

To analyze the predictive performance of the models, we have recently devised the *Cognitive Computation for Behavioral Reasoning Analysis* (CCOBRA) framework^1^. It has been used for recent model analyses in syllogistic (e.g., Brand et al., 2019, 2020; Dietz Saldanha and Schambach, 2020; Riesterer et al., 2020a,b), conditional (Ragni et al., 2019b), spatial reasoning (Ragni et al., 2019a; Todorovikj and Ragni, 2021), belief revision (Brand et al., 2021; Mannhardt et al., 2021), and models for fake news detection (Borukhson et al., 2021). It is driven by the idea that a model needs to face the same experimental setting and needs to be presented with the same reasoning problems in the same order as the participant received them. Consequently, the model given all the information, needs then to generate the participant's conclusion. If the model is able to predict this participant's conclusion, then the computational model is a model of that specific participant for that problem. As a consequence, if the model captures the cognitive processes of the participant accurately in the spatial domain, then we can expect a model to provide accurate predictions. If the predictive performance of a model is low, we can conclude that the theoretical accounts are inadequate representations of the cognitive reasoning processes.

To analyze the predictive performance of existing models, we need to take several steps: First, we need raw response data of each participant containing the specific spatial reasoning problem, how it has been presented, and which conclusion the participant has drawn. If there is variety in the type of spatial reasoning problems, e.g., by including data sets focusing on different types of relations such as cardinal directions (e.g., north, north-west, east) or one-dimensional relations (e.g., left and right), a comprehensive benchmark can be formed to analyze models' general ability to account for human spatial reasoning ability. Second, the method for model evaluation will rely on assessing how good the individual participant can be predicted by the models. To compute this, we will employ the predictive model evaluation settings provided by CCOBRA. Third, and maybe most importantly, implemented models for psychological theories of spatial reasoning are required. In particular, in order to evaluate their predictive ability, the implementations have to be able to provide precise predictions about the conclusions, experimental participants would infer. Finally, to evaluate a models' full predictive potential, it might be necessary to extend models in order to improve their ability to capture the inter-individual differences. For instance, if reasoning operates on the generation of mental representations of the premises, models may account for individual differences by either constructing none, some, or all possible alternative models. This flexibility allows them to decide which strategy is optimal for predicting responses given by a specific individual.

The paper is structured as follows: In the next section, an empirical section, we present the domain of spatial relational reasoning on selected problems and results. In section 3, a technical section, we will introduce the state of the art of cognitive models, their implementation and adaptation, and the predictive task we have used for their evaluation. Section 4 presents and discusses the results of our analysis and a general discussion about the predictive task in spatial relational reasoning concludes the paper.

## 2. Spatial Relational Reasoning Problems, Effects, and Benchmark

Example (1) in the introduction shows a typical spatial relational reasoning problem. This example consists of three terms, in this case cities, and two cardinal direction relations in-between them. In principle in those tasks, any spatial entity, such as objects on a table, geographical regions, or even stars can be in one or many infinitely relations such as left-of, contained in, above, and so on. In most experiments participants have either to generate a conclusion (*production task*) or to check if a possible conclusion holds (*verification task*). A production task requires more cognitive processes than a verification task, because at first a putative conclusion has to be found which needs then to be verified. In both cases, an initially constructed mental representation—the preferred mental model—may be key for explaining the specific conclusion inferred for the problem (for more background see, Ragni and Knauff, 2013).

A first psychological investigation of reasoning about spatial problems goes back to Störring (1908). Since then a broad variety of problems have been investigated. Among the most core and classical features count the *figural effect* (Ehrlich and Johnson-Laird, 1982), the *continuity effect* (Knauff et al., 1998), the *preference effect* (Knauff et al., 1995), and the *small-large scale-relations* effect (e.g., Potter, 1995), for more phenomena see Ragni (2008). In the following, we introduce these effects in greater detail. Additionally, since data sets collected for the investigation of these effects serves as the foundation for our analyses later on, we also describe the experimental settings in which they were obtained. As our focus is on individual responses, we do, however, not report aggregated statistics beyond the given responses. While it is desirable to have a broad range of different effects and raw data, the search was limited by the availability of raw data. Most studies have been conducted more than a decade ago and so in most cases only aggregated data have been available.

### 2.1. The Figural Effect

Consider the three-term problem “A is left of B” and “B is left of C”. There are four ways, called figures, that allow to reformulate this three-term problem using the relations *right* or *left* leading to the same spatial arrangement A-B-C. Table 1 represents these four figures, the formulation above with left-left is called Figure I, “A is left of B” and “C is right of B” is called Figure II and so on. Studies by Ehrlich and Johnson-Laird (1982) and Knauff et al. (1998) for spatial reasoning with interval relations show differences between the figures in performance.

**Table 1:** Term orders underlying the four different figures.

**Figure**	**Assertion 1**	**Assertion 2**
I	A ← B	B ← C
II	A ← B	C → B
III	B → A	B ← C
IV	B → A	C → B

*Please note that ← represents the relation “is to the left of” and → the relation “to the right of.”*

#### 2.1.1. Experimental Data—Procedure, Materials, Design, and Results

The first data set (*3ps.csv*)^1^ contains a total of 33 participants in an unpublished online Amazon's Mechanical Turk^2^ study conducted in 2011. Each participant received 16 problems. Each problem consisted of three spatial relational assertions, with the first two being Assertion 1 and Assertion 2 like in Table 1 with the variables being replaced by common fruit names. The third was a putative conclusion of the first and last objects. The participants received each assertion self-paced, which disappeared with the on-set of the next assertion. They had to press the keys “y,” for “yes, the set of assertions is consistent” and “n” for “no, the set of assertions is not consistent,” where a set of assertions is considered consistent if the putative conclusion does not logically contradict the other assertions. Out of the 16 problems, eight problems were consistent and another eight were inconsistent. The assertion reading times, response times, and given responses were recorded. The correctness of the participants was high (*Mdn* = 88%, *MAD* = 13), indicating that there were no major difficulties for participants in finding the logical response. All figures except of Figure III (75%, *SD* = 25) were solved by the participants correctly, indicating a non-measurable figural effect. Since the figural effect is relevant in syllogistic reasoning too, we still incorporate it in the benchmark for this analysis.

### 2.2. The Continuity Effect

The way information is presented can make it easier for participants to draw inferences (cp. Table 2). Consider the *continuous presentation* of each term in the following premise order “A is left of B” and “B is left of C” and “C is left of D.” Such problems are easier to process than semi-continuous problems, e.g., “B is left of C” and “C is left of D” where the third premise is: “A is left of B.” The second and the third premise have no term together, so the third premise can only be related via the term “B” to the first premise. The discontinuous problems present four different terms in the first two premises, which can accordingly not be related. Knauff et al. (1998) conducted an experiment to test the empirical differences between continuous, semi-continuous, and discontinuous orders of spatial assertions (cp. Table 2), following the general approach outlined in Ehrlich and Johnson-Laird (1982).

**Table 2:** The four-term-problems in the experiment by Knauff et al. (1998) with the reported error rates (in percentage correct).

	**Assertion**		
**Order of assertion**	**1**	**2**	**3**
Continuous	A–B	B–C	C–D
Semi-continuous	B–C	C–D	A–B
Discontinuous	C–D	A–B	B–C

*Participants were presented with colored intervals and had to specify all relations between A–D, A–C, and B–D. Please note that—represents a given interval relation that can be translated to “is to the left of” or “to the right of.”*

The study of Knauff et al. (1998) reports an increase in mean error rates from continuous (*M* = 39.7%), semi-continuous (*M* = 40.1) to discontinuous order (*M* = 50%). This effect has been replicated in Nejasmic et al. (2011).

#### 2.2.1. Experimental Data—Procedure, Materials, Design, and Results

The second data set (*4ps.csv*) contains a total of 30 participants, who received 24 consistent and 24 inconsistent problems, which are tested with the same sample as before from the same population as before. Each problem consisted of three assertions presented in continuous, semi-continuous, or discontinuous assertion order. The fourth assertion was a putative conclusion of the first and last introduced object in the premises. For about 25% of the problems, the conclusion could be rather easy falsified, as the converse of the conclusion was one premises. These problems served as a filter for participants, not deliberately trying to solve the problems but just randomly guessing or just following a heuristic considering the relation of the outmost terms. The participants received self-paced each assertion, which disappeared with the on-set of the next assertion. Hence, it followed the separate stage paradigm (Potts and Scholz, 1975). Participants had to press “y,” for “yes, the set of assertions is consistent” and “n” for “no, the set of assertions is not consistent.” For this analysis, no participants were excluded. The median correctness was high (*Mdn* = 73%, *MAD* = 15). The error rates were lowest for continuous (*Mdn* = 17%, *MAD* = 17), higher for semi-continuous (*Mdn* = 25%, *MAD* = 17), and highest for discontinuous assertions (*Mdn* = 33%, *MAD* = 8). The descriptive results are in line with the results reported in the literature.

### 2.3. Preference Effect

Reconsider the cardinal direction problem (1). What happens if more than one relation is possible? Are some preferred? This research question has been investigated for determinate and indeterminate problems. This effect has been identified for spatial relations as diverse as left and right (Ragni et al., 2007), for interval relations (Rauh et al., 2005), for topological relations (Knauff and Ragni, 2011), and for the eight cardinal directions (north, north-east, east, south-east, south, south-west, west, north-west; Ragni and Klein, 2012).

#### 2.3.1. Experimental Data—Procedure, Materials, Design, and Results

The third dataset (*carddir.csv*) contains 49 participants from an Amazon's Mechanical Turk study which was conducted in 2018 Ragni et al. (2019b). Participants received 64 spatial reasoning problems with cardinal directions. All problems were of the form “A *r*_1_ B. B *r*_2_ C.” with each *r*_1_ and *r*_2_ being one of the eight cardinal direction relations *north, north-east, east, south-east, south, south-west, west*, and *north-west*. Instead of A, B, and C different buildings based on their frequency in the English language were used.

The task for the participants was to give a relation that holds between C and A by selecting a conclusion out of the eight options obtained from combining the terms that only occurred in one of the premises by using one of the cardinal direction relations. The premises were presented sequentially in a self-paced procedure. The order of the problems was randomized separately for each participant. Participants responded by pressing the respective key/s (e.g., “nw” for north-west). Note that the task did not allow responding that all relations are possible, such as in the case of problems with opposite relations in both premises such as northwest and southeast, so the participants had a force-choice-task having to choose a preferred option instead. The results that in almost all cases participants preferred a relation can be found in Table 3.

**Table 3:** The aggregated preferred relation given by the participants for *C* − *A*, for the premises *Ar*_1_*B* and *Br*_2_*C* (Ragni et al., 2019a).

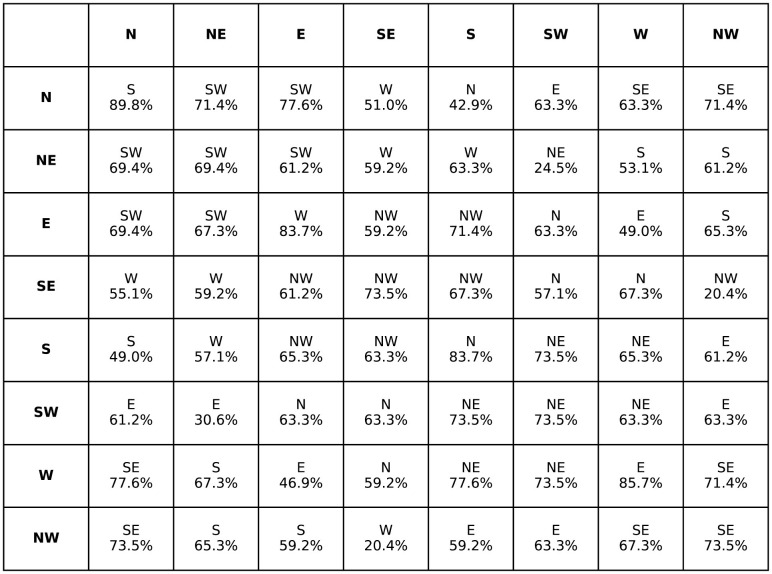

*Rows correspond to Ar_1_B, columns to Br_2_C*.

### 2.4. Small-Large-Scale-Relations Effect

This effect distinguishes between the construction principles for small-scale relations such as “left” which is used for scenarios of objects on tables or in rooms and large-scale relations such as the cardinal direction relation “West.” An example problem in the verification paradigm is as follows:

**Table:** 

(3) Left of the apricot is the pear.
Left of the pear is the fig.
Left of the pear is the kiwi.
Left of the kiwi is the cherry.
*Is the following arrangement a model of the premises?*
Fig Cherry Kiwi Pear Apricot [ff-strategy model]

for small-scale relations. Two other models for the premise are:

Cherry   Kiwi   Fig   Pear   Apricot     [f ff-strategy model]
Cherry   Fig   Kiwi   Pear   Apricot     [mix-strategy model]

The presented model can be constructed by the first-fit-strategy (Ragni and Knauff, 2013). This strategy is applied in premise 3 “Left of the pear is the kiwi.” in this case the kiwi is inserted in-between the pear and the fig, so on the first-fit position. The same is repeated with the cherry and the fig (premise 4). The first-free-fit strategy would have inserted the kiwi left of the fig. A mix strategy model combines both the ff and fff-insertion strategies. For large-scale relations instead of fruits, the fruit trees have been used.

#### 2.4.1. Experimental Data—Procedure, Materials, Design, and Results

The fourth dataset (*smalllarge.csv*) contains 51 participants that took part in a study in 2018 with the same sample as before. The first four premises introduce again information about the spatial relationship between terms. The experiment featured a set of four structurally distinct problems and used a set of 24 models to verify. Each of the problem-arrangement combination was presented twice resulting in a total of 48 problems for the participants to solve. The goal for the participants was to check their own mental representation of the relationships with the presented putative model and to decide whether it was consistent or inconsistent with the premise information. To make the model accessible in CCOBRA, however, it is encoded as a set of four assertions representing a relational model. The median correctness is 79% (*MAD* = 17), which is comparable to the experiments investigating the figural effect and the continuity effect. The preference effect is visible on the aggregate level, as not all models are equally likely accepted. The mean percentages are: for the first-free-fit strategy (small: 79%; large: 83%), the mix-strategy model (small: 74%; large: 72%), and the model constructed with the ff-strategy (small: 67%; large: 63%). This order fff ≥ mix ≥ ff holds on the individual level for 61% for small scale and 57% for large-scale relations.

## 3. Cognitive Models, the Predictive Task, and CCOBRA

### 3.1. Cognitive Models for Spatial Relational Reasoning

First conceptual models have been proposed in the second half of the 20th century (e.g., Mani and Johnson-Laird, 1982), first implemented theories have been provided about 30 years ago (Johnson-Laird and Byrne, 1991; Rips, 1994). While there exists a large variety of models for spatial reasoning (for an overview see Friemann and Ragni, 2018), most models have been assessed so far only on the reproducibility of effects such as the four presented above. Few of the existing cognitive models have been tested on predicting individual data. In Ragni et al. (2019a), five cognitive models for spatial reasoning were analyzed on the cardinal direction data set: The Spatial Probabilistic Model (Ragni and Becker, 2010), Verbal Spatial Reasoning Model (Krumnack et al., 2010), the Spatial Artificial Neural Network (Ragni and Klein, 2012), PRISM (Ragni and Knauff, 2013), and the Dynamic Field Theory (DFT, Kounatidou et al., 2018). The models demonstrated a similar predictive performance. In the following analysis, we focus on mental model approaches and apply the selection criteria from Ragni et al. (2019a): (i) the cognitive model has been developed for general spatial reasoning, (ii) the model already has an implemented version or is easily implementable, (iii) the model offers explanations for basal principles of spatial reasoning. This includes the model-based approaches PRISM and an extension of the Verbal Spatial Reasoning Model that have demonstrated highest performance on the cardinal direction data. A third model, the Spatial Reasoner (Johnson-Laird and Byrne, 1991) satisfies these criteria too.

#### 3.1.1. SpatialReasoner

The SpatialReasoner, a Lisp program called “space-6”^3^, is an implementation of the mental model theory for spatial relational reasoning (Johnson-Laird and Byrne, 1991). The program assigns to each term in a spatial premise a three dimensional Cartesian coordinate. For indeterminate spatial descriptions, it asserts two spatial objects in the same place “temporarily in the course of searching for models that refute conclusions.” as described in the source code of the algorithm.

Consider the premises “The A is to the left of the B” and “The C is to the right of the B.” The first premise will result in the creation of a new model with “A” on the left side of “B.” Hence, the coordinates of “A” will be assigned to the default coordinates of (0, 0, 0). “B” is on the right side of “A,” so the coordinates of “B” are (1, 0, 0) in the model. With the second premise, “C” will be inserted into the existing model. The program searches the first free coordinates that satisfy the relation between “B” and “C.” This results in “C” being inserted into the model at coordinates (2, 0, 0).


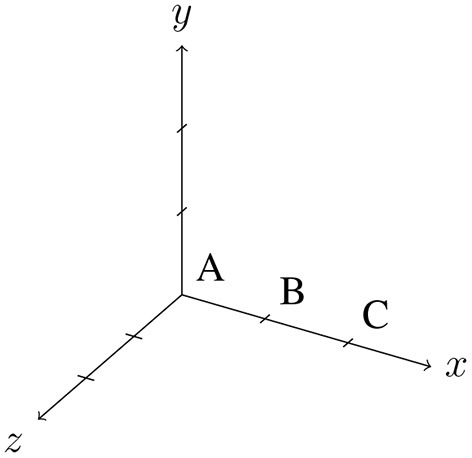


With these premises, an initial mental model is constructed. In a second step it is inspected to search for implicit relation, like “A is left of C,” which is called an initial or putative conclusion. For indeterminate problems (e.g., Example 1 above) alternative models can be searched for to falsify the previous conclusion.

The LISP-based implementation has been extended to account for different spatial relations, different objects, and to be able to predict an individuals conclusion to be compliant with the CCOBRA evaluation framework^4^. The SpatialReasoner original purpose was to integrate premises into a mental model that is then returned and to check conclusions. The SpatialReasoner, however, provides information about the success of integrating a new premise. SpatialReasoner checks the consistency of the new premise in terms of the mental model constructed thus far. While integrating a new premise into given premises, we extracted and interpreted the following cases from SpatialReasoner:

1. *Truth*, a new premise follows validly from the previous premises.
2. *Falsity*, a new premise is inconsistent with the previous premises.
3. *Weak falsification*, the constructed model was previously true.
4. *Weak truthification*, the constructed model was previously false. However, by reinterpreting the previous premises, a mental model consistent with all premises, including the new one, is found.

Interpreting these assertions about the model-based validity of premises allows us to transform SpatialReasoner into a predictive model. Our Python-based SpatialReasoner model directly communicates with the original LISP-based SpatialReasoner implementation and thus ensures that its predictions are still in line with the original authors' intentions. To extract SpatialReasoner's assertion, it first concatenates the problem's premises with the putative conclusion in order to arrive at a problem formulation consisting of a series of premises for SpatialReasoner to attempt to integrate into a single mental model. Extracting the assertion about the problem's validity from the LISP-output, our model then proceeds to interpret it for generating the binary prediction about validity or falsity of the problem. To do so, we implemented four model variants representing possible individual characteristics:

- *SpatialReasoner-skeptical* considers only conclusion candidates necessarily following from the premises, i.e., corresponding to the first of SpatialReasoner's validity assertions introduced above.
- *SpatialReasoner-credulous* is located at the other end of the spectrum and accepts all conclusions that can possibly follow, i.e., all of SpatialReasoner's assertions except for the second one.
- *SpatialReasoner-initial* only considers conclusions as true that initially appeared valid, i.e., SpatialReasoner's first and third assertion.
- *SpatialReasoner-adapted* represents an individualized model that selects the variant fitting best to a participant's responses.

#### 3.1.2. PRISM

**Figure 1:**
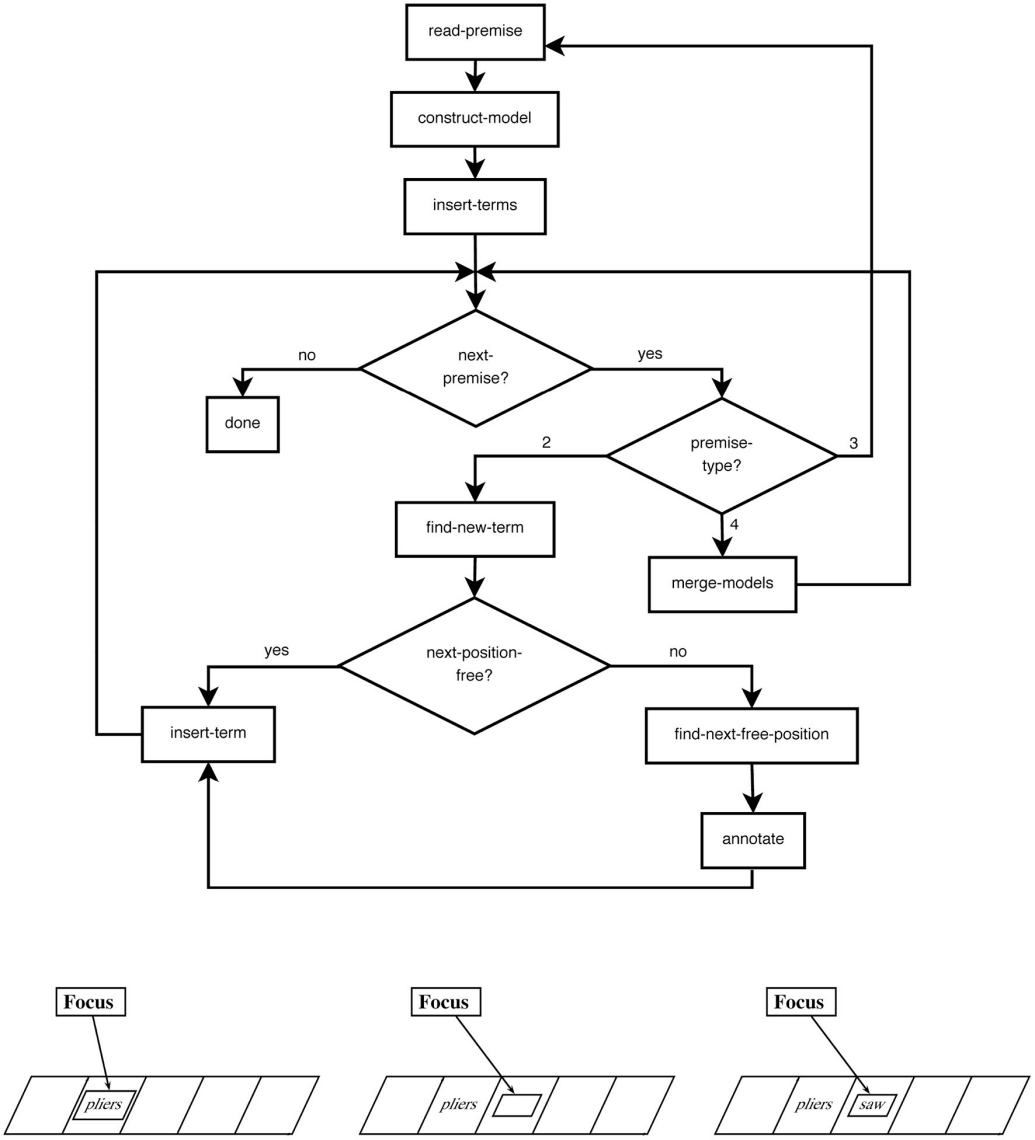
PRISMs model construction process demonstrated on the example premise “the pliers are to the left of the saw.”

PRISM is the implementation of the preferred mental model theory (Ragni and Knauff, 2013) and simulates human performance in spatial reasoning tasks. It consists of a spatial working memory structure operationalized by a spatial array and a spatial focus which inserts spatial objects into the array, inspects the array to find new spatial relations, and, if necessary, relocates spatial objects. It consists of four parts: *I*, the input mechanism, which reads the premises from an external device, a set *O* of object names, a spatial focus *F*, that operates on a spatial array and can move right, left, forward, and backward, and *C*, the control process, which is responsible for the control of the focus and other executive functions. Let us consider the example premise “The pliers are to the left of the saw.” PRISM assumes a discrete space representation, which can be represented by a multidimensional array and constructs a mental model successively by inserting one object after the other by using a focus. Hence, the focus that is initially at position (0,0) inserts an icon of “the pliers” and by processing the next part of the premise, moves to the right and inserts an icon of “the saw.” Figure 1 provides PRISM's control process and a visualization of constructing a mental model out of the given premises.

For indeterminate problems like (1) above, it constructs the most parsimonious mental model of the premises given the number of operations of the spatial focus. Depending on the premise type, it integrates the terms of the premise accordingly (see Figure 1). This model is called the preferred mental model. The model construction principles differ from the initial model the spatial reasoner generates and so different putative conclusions are drawn. The preferred model is varied according to minimal changes (cp. Ragni and Knauff, 2013) to find alternative models. Conclusions are generated or verified based on the preferred mental model. This behavior may lead to logically incorrect answers and fits well to typical errors and performance rates of human subjects (Ragni et al., 2007; Ragni and Brüssow, 2011). The focus introduces a general measure of difficulty based on the number of necessary focus operations (rather than the number of models). Individual differences can be explained by differences in the construction of the preferred mental model and, if and how many, alternative models are taken into account.

#### 3.1.3. Verbal Spatial Reasoning Model

The Verbal Spatial Reasoning Model (Krumnack et al., 2010, 2011) suggests that individuals construct a queue of spatial objects in their mind. They distinguish between the “general structure of verbal models and the most efficient process of constructing these models” (Krumnack et al., 2011, p. 379). It is understood as an operationalization of mechanisms of language processes as proposed by Polk and Newell (1995). The most efficient process is determined by a *mental cost metric*. This metric is defined based on the insertion, breaking of, and searching in the direction of links between spatial objects: breaking a link costs more than creating one, and searching has a culturally dependent left-right preference (e.g., Maass and Russo, 2003).

As an example, consider the following premises (taken from Krumnack et al., 2011):

The apple is to the left of the mango.
The apple is to the left of the pear.

and the conclusions “The mango is to the left of the pear” and “The pear is to the left of the mango.”

The main assumption of the verbal reasoning approach is the existence of a “queue” of objects. Thereby the queue describes the order of the object, but the interpretation of the order depends on the respective relation at hand (e.g., it can be an order by size, value, position, etc). The queue is constructed by creating directed links between the objects, which allow to traverse the queue from the beginning in order to access the objects. This leads to the following rules for the construction:

1. The first object inserted in the queue, is the starting point of the queue.
2. The second object is linked to the first object. The relation determines the interpretation and the implicit direction of all following objects.

By applying these rules, two possible models can be constructed from the first premise:

apple* → mango
apple ← mango*

where the asterisk (*) denotes the starting point. For the second premise, the object “pear” has to be inserted. There are two rules for the insertion (Krumnack et al., 2010, 2011):

1. If the new object is to be placed behind an object of the queue it will be inserted at the end of the queue.
2. If the new object is to be placed in front of an object of the queue it will be inserted into the queue directly in front of this object.

The application of the rules lead to the following possible queues:

apple* → mango → pear
apple ← pear ← mango*

To decide which of the possible models are preferred, the cognitive cost of each model is estimated. For the first model, a new link has to be created in order to connect “pear” to “mango.” For the second model, the already existing link between “mango” and “apple” needs to be removed in addition to connecting “pear,” leading to higher overall costs. For both models, however, a different conclusion can be followed: “The mango is to the left of the pear” follows from the first model, while the second conclusion “The pear is to the left of the mango” follows from the latter. It was shown that the first conclusions was easier to accept, which indicates that the model with lower cognitive cost was indeed preferred (Krumnack et al., 2011).

As the model by Krumnack et al. (2010) has been developed for one-dimensional spatial relational problems only (e.g., left-right), it has been extended for spatial reasoning with cardinal directions by combining the different dimensions in a Cartesian coordinate system way (Ragni et al., 2019a): For the vertical and horizontal plane respectively a direction encoding is added to each link, with positive values for “north” and “east,” and negative ones for “south” and “west.” If the angle between the direction of the new relation and the queue direction is >90°, the new object is inserted before the reference object, otherwise at the end of the queue. To predict a response the model sums up all the direction encodings between the two objects in the queue and decodes them into cardinal directions.

Each presented computational model shares key features with the other models: They all build mental models and there are similar processes for model construction, inspection, and variation. But the models differ in some aspects: PRISM has specific processes on constructing a preferred mental model in contrast to the SpatialReasoner, the VerbalReasoner differs that objects are linked and there is a preferred order. Finally, the SpatialReasoner (cp. the variants we describe above) allows for differences in the inference mechanisms. A comparison on the individual participant allows to have a fine-grained analysis, i.e., which strategies of constructing a model, or which inference mechanism is used by this specific person.

### 3.2. The Predictive Cognitive Modeling Task

The goal of the evaluation presented in this paper is to investigate the predictive capabilities of the current state of the art in modeling spatial relational reasoning.

Our analysis is based on the *coverage task* (Riesterer et al., 2020a), an evaluation setting in which models are assessed based on their ability to predict the specific responses given by participants in the experimental setting. In doing so, models are evaluated based on their ability to accurately reflect the human reasoning processes. Crucially, in the coverage setting, the models are provided with the exact problem-response data they are subsequently supposed to predict beforehand.

**Figure 2:**
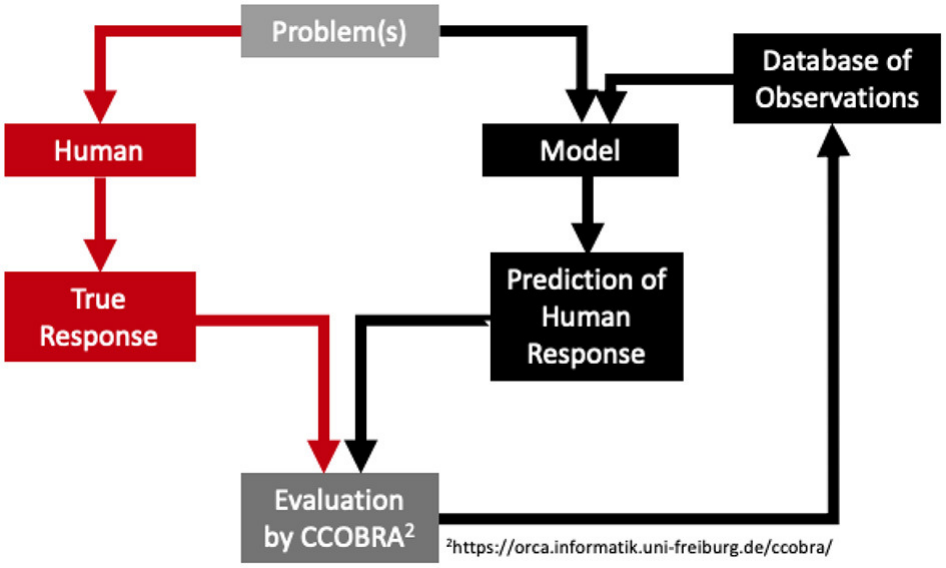
The CCOBRA-framework to evaluate the predictive power of cognitive theories.

To analyze model performances in a standardized fashion, the *Cognitive Computation for Behavioral Reasoning Analysis* (CCOBRA) framework is used^1^. CCOBRA is a framework (for an overview over its core flow, see Figure 2), which enables the comparison of cognitive models in various domains including the spatial relational domain. One of the main goals of the framework is to evaluate models with respect to their ability to predict actual reasoning behavior observed in psychological experimentation, i.e., on the level of individual responses. Hence, the data required by CCOBRA are experimental problems and the corresponding participant responses. In a training phase, models are given the possibility to search for a parameter configuration that optimally captures the individual reasoner to predict. Consequently, if a reasoner's response behavior fits into the configuration space spanned by the model, it is able to reproduce, or “cover,” the reasoner's behavior. If, however, the model is unable to provide an adequate parameterization capturing the reasoner's behavior, it will provide inaccurate response predictions and thus receive a low coverage score. Because substantial inter-individual differences are to be expected in reasoning behavior, CCOBRA allows models to rely on three different types of fitting functions in order dynamically tailor their inferential mechanisms to specific reasoners.

First, *pre-train* provides a set of experimental data (i.e., problems and their corresponding responses) that excludes the participant for which predictions are to be generated. This data can be used by the model to adjust itself prior to the actual evaluation. This resembles the training phase of modern machine learning methods. The later prediction phase then tests the model's ability to generalize from the training examples to novel reasoning behavior. Second, *pre-train-person* is a second pre-training phase that occurs before the prediction phase in which exemplary data for the individual is supplied to be predicted for. In doing so, the model is provided with a first glance at the behavior it is subsequently expected to reflect. Finally, an *adaption* phase is performed after a prediction has been generated by the model. In this phase, a model is provided with the true response to the problem it has just generated a prediction for. This allows models to continuously fine-tune their inferences to match an individual's behavior as closely as possible. Since our analyses are based on the coverage setting, only pre-train and pre-train-person are used. Importantly, during the pre-train-person phase, models are provided with the exact problem-response pairs they are subsequently expected to predict. This allows them to determine an optimal parameterization for the individual.

Since CCOBRA aims at simulating the experimental setting, models need to be able to provide precise response predictions. In particular, this means that if a model is based on probabilistic principles, a singular response needs to be determined similar to the human reasoner who might be forced to select a singular response even if they are unconsciously assessing the likelihood of different response options to be valid. However, this focus on precise response predictions allows CCOBRA to evaluate arbitrary models irrespective of their formal foundation.

CCOBRA approaches model evaluation from the perspective of benchmarks. Given experimental datasets consisting of problems and responses, CCOBRA allows for an evaluation of cognitive model performance in a standardized manner. At the end of the evaluation, the results, i.e., the obtained response predictions as well as corresponding accuracy scores are returned in conjunction with visualizations representing the model performances.

## 4. Results

In this section we put the different cognitive computational models to the test. In addition to the cognitive models, five baseline models are introduced to provide reference performances that allow a better interpretation of the cognitive model performances. First, the *RandomModel* generates responses by randomly selecting one of the possible responses, i.e., true or false in verification tasks or one of the response choices in production tasks. In doing so, it serves as a lower baseline for all models that claim to incorporate insight into spatial reasoning processes should exceed. Second, the *Most-Frequent Answer* model (MFA) generates predictions by selecting the response most frequently selected by the participants in the training data. This means that the MFA serves as an upper bound of performance for models that do not incorporate inter-individual differentiation and thus dynamicity in their response behavior. Third, the *TransitiveClosure* model is a purely logical model that generates all possible relations consistent to the premises by repeatedly applying the transitive rule to the relations obtained thus far. Note that for verification tasks that only ask participants to validate a conclusion, the performance of TransitiveClosure will just reflect the correctness of the participants' responses. However, when multiple correct response options are available, the model performance drops, as it will select one of the correct options at random. Finally, we included two models, *BestModel* and *Optimal* that artificially attempt to provide an estimate of the optimal performance achievable by the current cognitive models. To this end, *BestModel* selects the optimal cognitive model for each participant and uses it to generate predictions. *Optimal* determines the best cognitive model on the level of individual problems, which means that it generates the correct prediction for a problem if at least one of the cognitive models is able to generate the correct prediction.

### 4.1. Data Set 1: The Figural Effect

**Figure 3:**
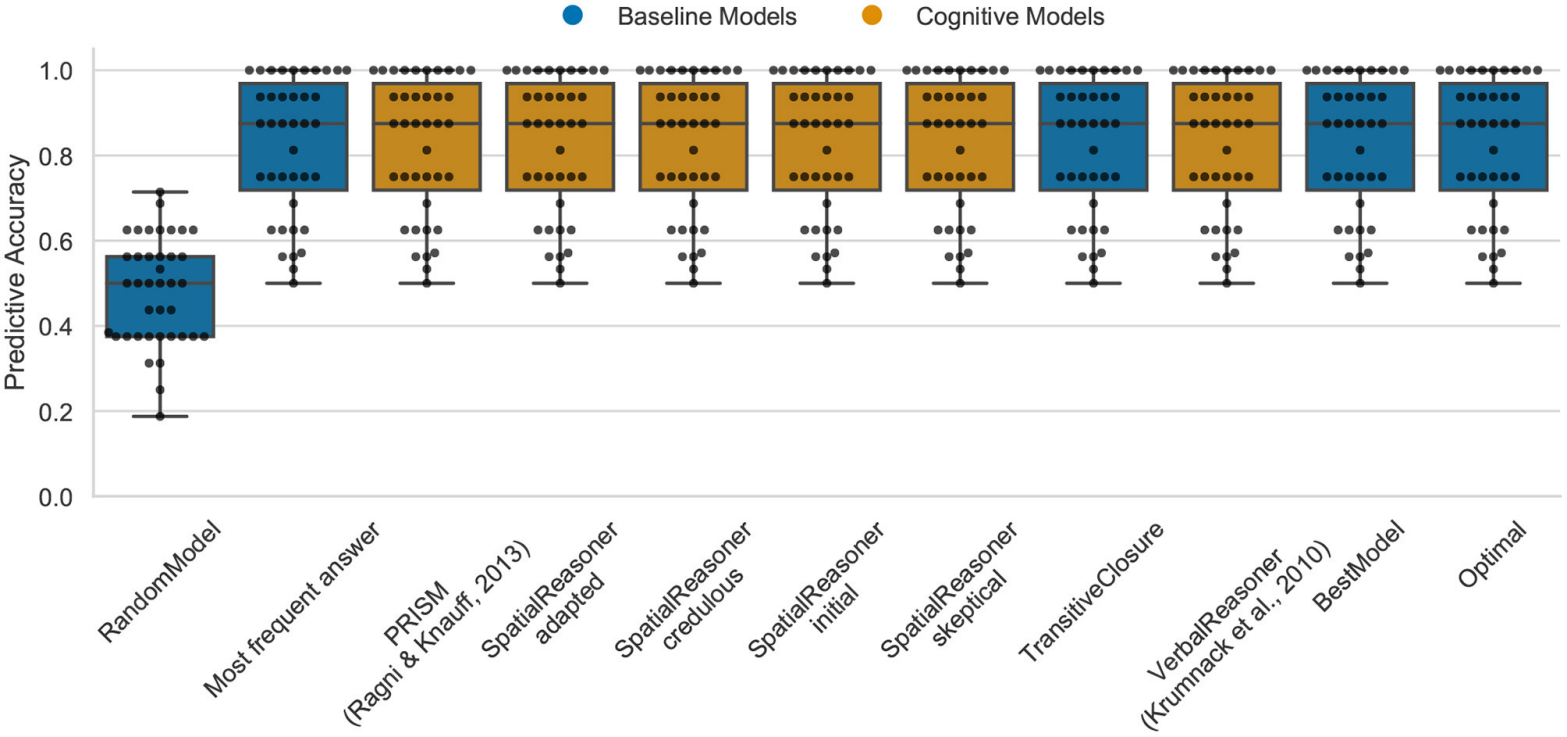
The predictive performance of the cognitive computational models and baseline models on the figural effect data set (*3ps.csv*). Predictive accuracy is defined as the ratio between the number of correct predictions of individual participants' responses and the total number of predictions. Dots represent the predictive accuracy for individual participants.

In the case of the figural data, the Optimal model is able to predict about 88% (median) of the participants responses (cp. Figure 3). The performance of the models is except of the RandomModel identical (cp. Figure 3). In fact, comparing the results with the TransitiveClosure demonstrates that all existing models do replicate the logical correct answer. This shows that the difference among the models does not contribute anything to explain inter-individual differences. This indicates that there are still cognitive processes, being either reasoning or guessing principles, that are not captured by models. However, the good performance of the MFA also indicate that the participants do not show a lot of variance in this task, with the majority of participants also giving the logical correct answer. Therefore, the task seems to be too easy to allow a differentiation of the models, as the results are mostly dominated by the correct answer.

### 4.2. Data Set 2: The Continuity Effect

**Figure 4:**
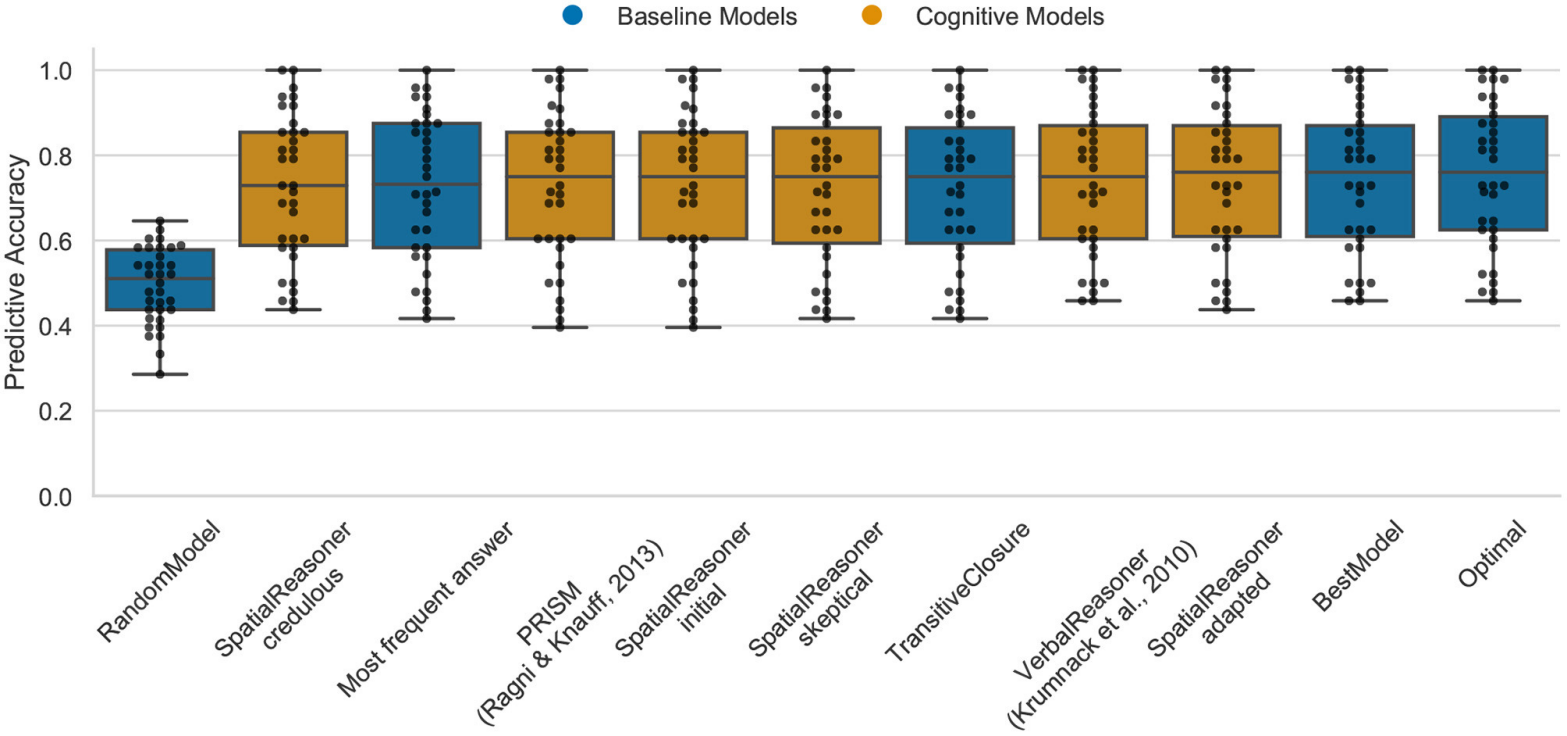
The predictive performance of the cognitive computational models and baseline models on the continuity effect data set (*4ps.csv*). Predictive accuracy is defined as the ratio between the number of correct predictions of individual participants' responses and the total number of predictions. Dots represent the predictive accuracy for individual participants.

The Optimal model shows the upper bound for the predictive task of about 76% (median) for the continuity data (cp. Figure 4). The performance of the models is, except of the RandomModel, again very similar. SpatialReasoner-adapted has the highest performance with a median of 76% with PRISM, VerbalReasoner, SpatialReasoner-skeptical, SpatialReasoner-initial, and TransitiveClosure with 75%. In this case, the models again do most often just predict the logical responses given by the participants. This indicates that there are still cognitive processes, being either reasoning or guessing principles, that are not captured by models. This and the previous data set are not distinguishing between the models, they are not diagnostic in the sense that they can enlighten underlying differences of the models.

In a next step, we analyzed the predictive performance of the cognitive models with respect to the subcondition continuous, semi-continuous, and discontinuous, but the performance was identical.

### 4.3. Data Set 3: The Preference Effect

**Figure 5:**
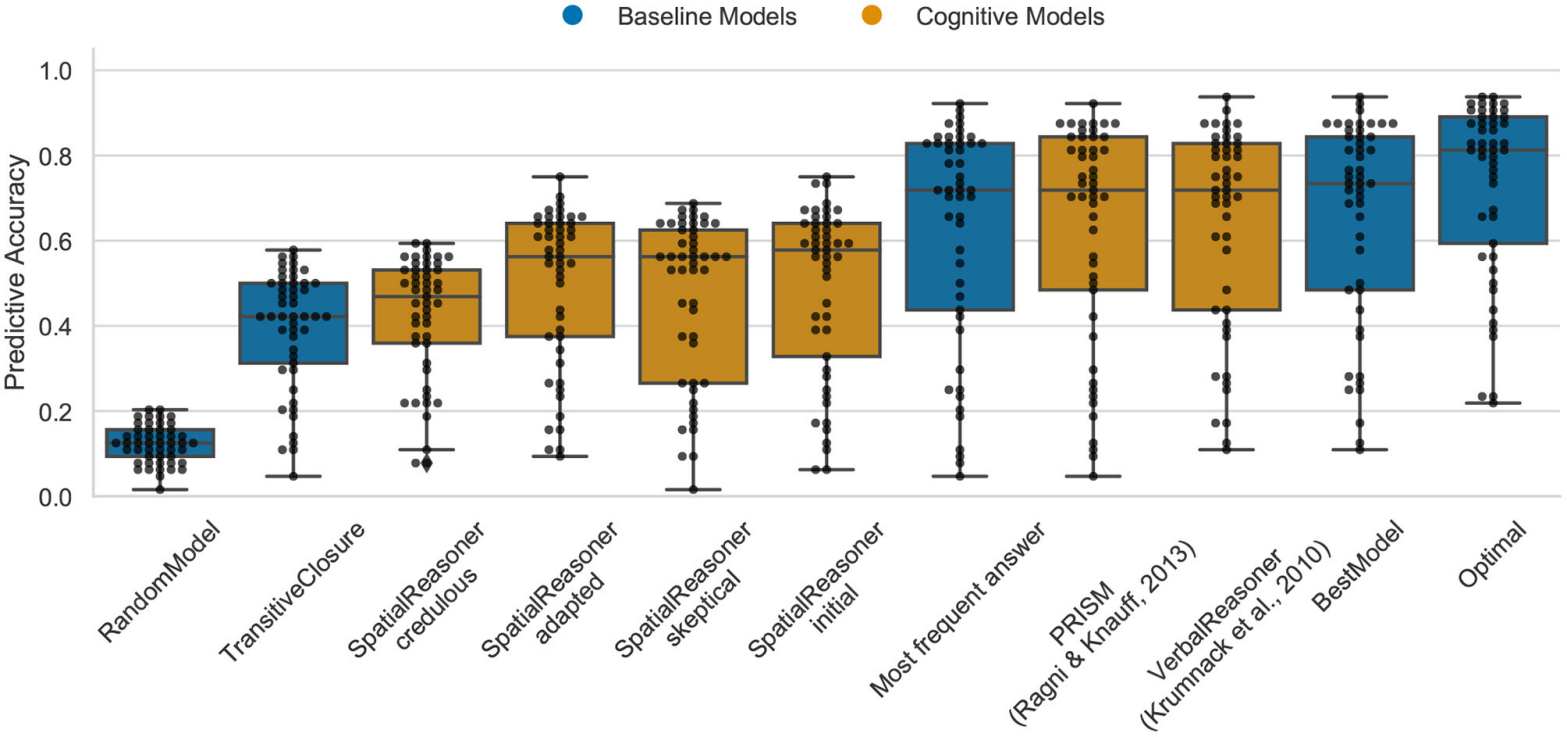
The predictive performance of the cognitive computational models and baseline models on the preference effect data set (*carddir.csv*). Predictive accuracy is defined as the ratio between the number of correct predictions of individual participants' responses and the total number of predictions. Dots represent the predictive accuracy for individual participants.

The Optimal model shows again the upper bound for the predictive task as 82% (median) for the preference effect for cardinal direction (cp. Figure 5). In this case the “oracle” clearly outperforms the cognitive models. The preference effect in the cardinal direction shows more variation in the models, the best performance is demonstrated by PRISM and VerbalReasoner which both achieve 71.9%. Both models, PRISM and VerbalReasoner differ in their predictions. This explains why both contribute to the higher performance of the Optimal model. The SpatialReasoner does not perform as well, SpatialReasoner-initial (with 57.1%) indicating that the SpatialReasoners model construction process does not yet capture the preference effect in reasoning with cardinal directions.

### 4.4. Data Set 4: The Small-Large-Scale Relation Effect

**Figure 6:**
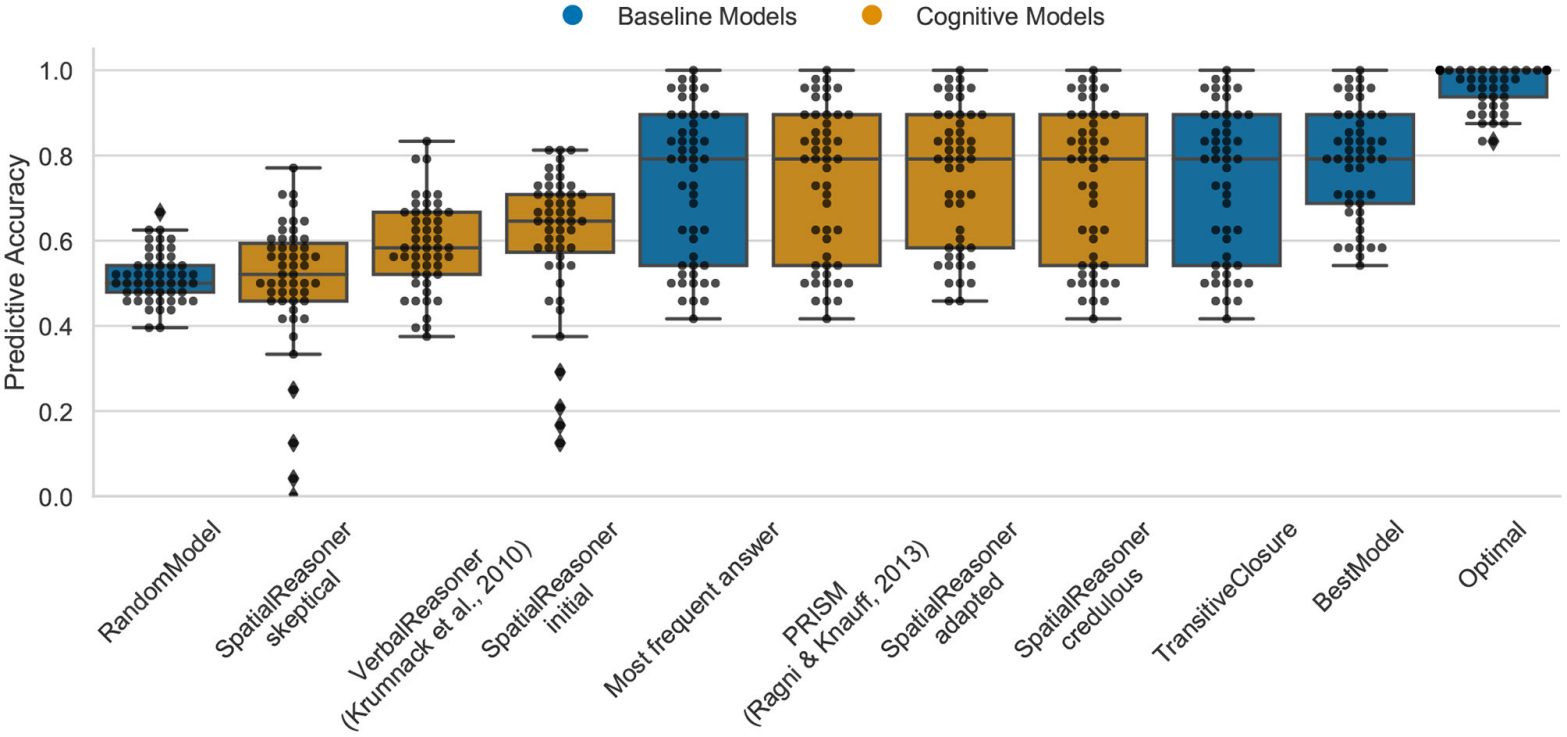
The predictive performance of the cognitive computational models and baseline models on the small-large-scale relations effect data set (*smallarge.csv*). Predictive accuracy is defined as the ratio between the number of correct predictions of individual participants' responses and the total number of predictions. Dots represent the predictive accuracy for individual participants.

The Optimal model again shows the upper bound for predictive task as 100% (median) for the small-large-scale relation effect (cp. Figure 6). This demonstrates that the existing cognitive models already capture most of the cognitive processes participants apply. The highest performance of 79.1% in between the Optimal model and the MFA show PRISM, SpatialReasoner-adapted and -credulous. It is notable that VerbalReasoner drops from its previous high performance which was often close to the best models in the other benchmark data. The TransitiveClosure baseline model ranges at 73.4%, indicating that participants showed a high dependency on logics in this task.

### 4.5. The Contribution of the Cognitive Models to the Optimal Model

**Figure 7:**
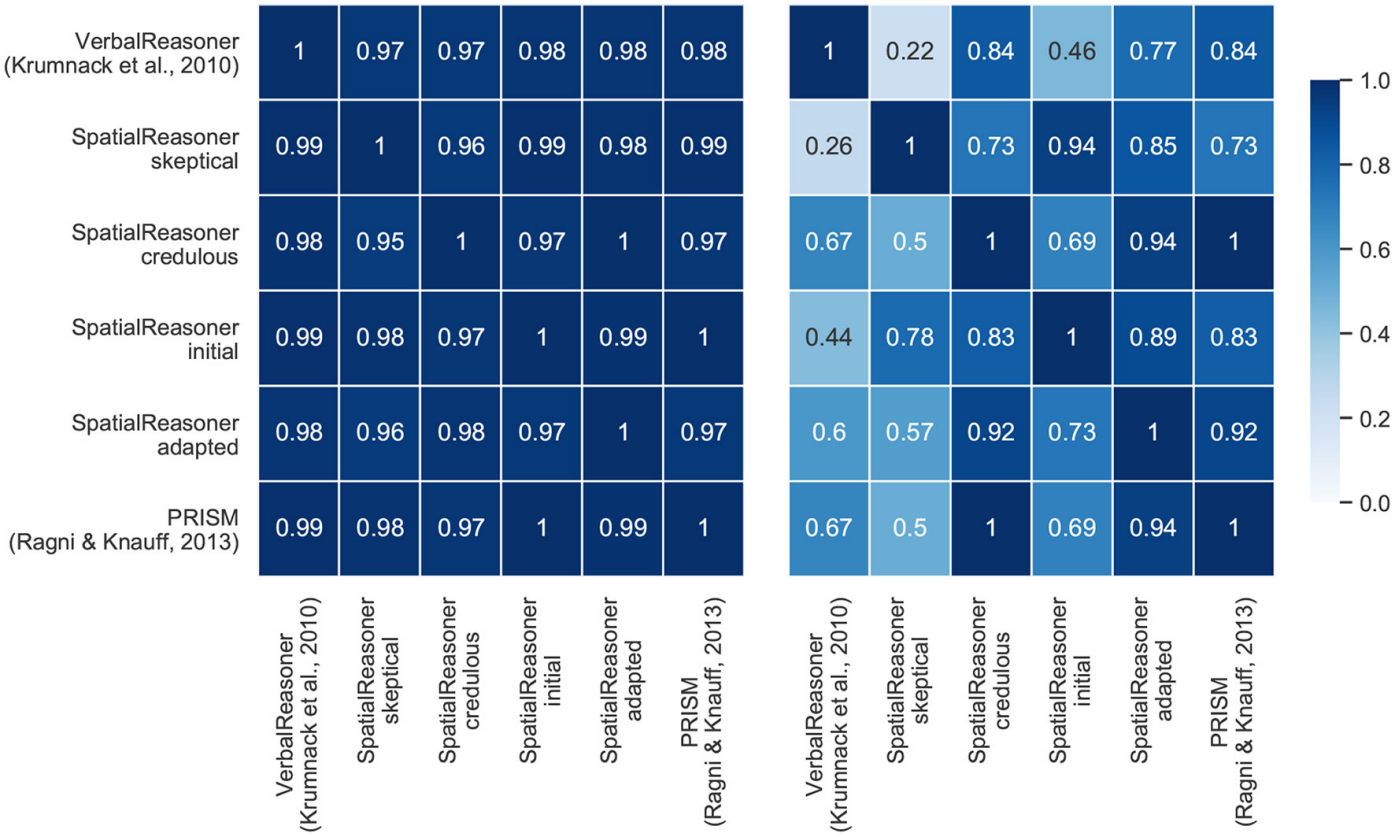
Heatmap that visualizes how often the models were optimal together and for the same problem (left: Data set 2—continuity effect; right: Data set 4—Small-Large-Scale). Note that the heatmaps are not symmetrical: if the model of the current line was optimal, how often was the model in the respective column also optimal.

Data set 2 (the continuity effect) and data set 4 (small-large-scale relation) differ with respect to the models' contribution to the optimal model as can be seen in Figure 7. The heatmap for data set 2 shows that almost all models contribute the same amount of conclusions to the optimal model. This is different for data set 4. PRISM and SpatialReasoner-credulous have a high congruency as can be seen in the heatmap (cp. Figure 7). In contrast, SpatialReasoner-skeptical provides the most distinctly different set of responses. This indicates that both approaches provide predictions that are able to accommodate different parts of the behavioral patterns contained in the data, i.e., the varying cognitive processes in human reasoners.

## 5. General Discussion

Cognitive modeling has so far focused on explaining group-level data such as the aggregated responses of individuals. How good existing computational models for spatial reasoning can predict the respective conclusions of any individual reasoner is unknown. The goal of this article is to focus on the individual reasoner. We identified and analyzed first data sets for the inclusion in a benchmark, we adapted and individualized existing computational models, and evaluated the models on the data sets. The models were evaluated on spatial relational descriptions. The task of the participants was to process the spatial information and to produce or verify a conclusion or possible mental model.

Refined cognitive models demonstrated a predictive power for any individuals conclusion of about 80% (median) across data sets. Data sets that include more complex effects with respect to the cognitive processes and tasks, such as the preference and small-large-scale relation effect were able to better differentiate between the models than information presentation effects, than the continuity or figural effect. In fact, for both problems the tasks were so easy that an instantiation of a logical model (TransitiveClosure) performed identical. Hence, not only more data and effects for developing a benchmark is necessary, but data in which participants significantly deviate from logical approaches are required. The Heatmap in Figure 7 demonstrates that for the Small-Large-Scale data set the three models SpatialReasoner (with its variants), PRISM, and VerbalReasoner, despite being developed in that order, can differ significantly. Especially enriching models such as the SpatialReasoner with different inference mechanisms can lead to a large difference in their predictive performance (cp. Figures 6, 7). Given that different individuals can apply different (weak) inference mechanisms, this allows to individualize models better.

This fine-grained analysis of the features were only possible by considering each individual responses of each participant. It eliminates the preprocessing of data and goes beyond a pure aggregated statistical evaluation. Aggregation can help to identify general effects, at the same time it does not guarantee that these general effects can be found in an individual (Kievit et al., 2016; Fisher et al., 2018).

### 5.1. What Are Limitations of the Benchmark Set?

Overall, there are only marginal differences between models for the first two data sets, and so models cannot be evaluated meaningfully. From a benchmark and modeling point of view, the data set is not diagnostic. Nothing changes in evaluating subconditions in the continuity effect: there are only slight differences between the performance within models, most models behave very similar. It is different with the Small-Large-Scale Relation dataset, e.g., VerbalReasoner in particular behaves differently than the other models. This, however, is not due to the small-scale and large-scale effect. Evaluated based on each models' respective contribution to the Optimal model, it shows that high performing models (e.g., SpatialReasoner-credulous) outperform models such as VerbalReasoner on both subconditions. It would have been desirable to have more and larger data sets, but raw data of most publications that date some time back is not yet accessible. If the tasks require more than the acceptance or rejection of a conclusion (conclusion-verification tasks), but to generate many possible responses (like in cardinal directions), then a higher discrimination rate between models is possible. Hence, conclusion generation tasks with several possible conclusions is—from the perspective of computational model comparisons—preferred over conclusion verification tasks with a dichotomous response set.

### 5.2. What Are Limitations of the Existing Cognitive Models?

The first two data sets analyze the impact the presentation of spatial information has on the way how humans generate conclusions. Assessing the cognitive models demonstrate their congruence with classical logic, i.e., they do not contribute more explanation power than logic. This indicates that existing models need to be extended with more cognitive processes to capture more of the given responses of participants. Additionally, given the uniformity in the models' prediction on these data, it seems that more research effort is necessary to understand why and how humans deviate in these rather simple tasks from logics. This changes with the preference effect and smalllarge data set. Here the models differ more and different strategies lead to a better assessment of the individual model contribution. So these data sets are suited better to capture the indeterminacy in the models. The cognitive processes in models are not yet fully modularized. To built more adaptive and dynamic models it is an advantage to identify atomic cognitive operations (cp. for syllogistic reasoning, Bischofberger and Ragni, 2021).

So far many publications in the psychology of spatial reasoning have focused on reporting general effects on the aggregated level. But a core result of our analysis is that not all identified effects in psychological experiments are helpful in evaluating models. So far, many model's prediction are congruent with each other in explaining effects, such as the figural and continuity effect. From a modeling perspective, this either indicates that many models are similar, that core effects are not suitable for separating different models well. A further point is, as the figural effect demonstrates, that sometimes individuals do not differ on tasks. The current approach, that we need to identify more and more effects experimentally remains unclear—as there theoretically can be infinite many possible effects. In turn, we argue for a different approach, that we have outlined here: We propose that future models need to focus more and more on predicting any individual's conclusion. This avoids illusions of effects due to aggregation, gives a precise understandable measure of how good a model performs, allows to compare and reject models based on how good they just predict this individual's conclusion, and offers the possibility to identify missing cognitive processes of models that are not yet considered in models. The predictive modeling task calls for a further joint effort and true interdisciplinary approach where the best of cognitive psychology, machine learning, and classical AI are combined to predict and understand *why* and *how* an individual derives a conclusion.

## Data Availability Statement

The model scripts and data can be found in the reposity: https://github.com/CognitiveComputationLab/2020-spatialmmt.

## Ethics Statement

Ethical review and approval was not required for the study on human participants in accordance with the local legislation and institutional requirements. Written informed consent for participation was not required for this study in accordance with the national legislation and the institutional requirements.

## Author Contributions

MR has written the paper with contributions by DB and NR. Data and some models have been provided by MR. NR and DB adapted and prepared SpatialReasoner and the baseline models as well as performed the model evaluation via CCOBRA. All authors have discussed and analyzed data and models.

## Funding

This paper was supported by DFG grants RA 1934/9-1, RA 1934/10-1, and RA 1934/4-1, and a DIAS-fellowship by the SDU to MR. The publication of this article was funded by Chemnitz University of Technology.

## Conflict of Interest

The authors declare that the research was conducted in the absence of any commercial or financial relationships that could be construed as a potential conflict of interest.

## Publisher's Note

All claims expressed in this article are solely those of the authors and do not necessarily represent those of their affiliated organizations, or those of the publisher, the editors and the reviewers. Any product that may be evaluated in this article, or claim that may be made by its manufacturer, is not guaranteed or endorsed by the publisher.

## Footnotes

^1^ https://github.com/CognitiveComputationLab/ccobra

^2^ http://www.mturk.com

^3^ http://modeltheory.org/programs/space-6.lisp

^4^Source code of the implementation: https://github.com/nriesterer/pyspatialreasoner.
